# ‘All Ears’: A Questionnaire of 1516 Owner Perceptions of the Mental Abilities of Pet Rabbits, Subsequent Resource Provision, and the Effect on Welfare

**DOI:** 10.3390/ani10101730

**Published:** 2020-09-23

**Authors:** Sarah A. McMahon, Ellie Wigham

**Affiliations:** School of Veterinary Medicine, College of Medical, Veterinary and Life Sciences, University of Glasgow, Glasgow G61 1QH, UK; 2178174M@student.glasgow.ac.uk

**Keywords:** rabbit, welfare, perception, survey, behavior, human-animal interactions, resources, pet

## Abstract

**Simple Summary:**

Recent research has found that pet rabbits are frequently inappropriately housed, fed, and not routinely provided with healthcare, often suffering painful medical conditions and shortened lifespans as a result. Using an online survey, this study provides a new understanding of rabbit owners’ perceptions of rabbits, how these affect the resources (e.g., diet) that rabbits are provided with, and how these resources impact rabbit welfare. Our results found that variation in owner perception influenced provisions of conspecific partners, level of enrichment, diet, and type of housing provided. Welfare scores (produced from owner-reported behavioral frequencies) were improved with a diet of mainly grass/hay, a larger variety of enrichment, free-roam housing, and increased time spent with owners. These results suggest that a practical approach to improving the welfare standard provided to rabbits may be to target improving owner perceptions. This information would be beneficial in tailoring public education programs to increase provision of welfare enhancing resources, improving the human–animal relationship, and improving the welfare standards for rabbits.

**Abstract:**

Pet rabbit welfare is a hidden crisis: Inappropriately housed, fed, and not routinely provided healthcare—rabbits can often suffer painful conditions and shortened lifespans. This study provides novel understanding of owners’ perceptions of rabbits’ mental capabilities; how this impacts their husbandry; and subsequent effects on rabbits’ welfare. A survey was designed to investigate owner and rabbit demographics, owner perception of rabbits, resources provided, and rabbit behavior. Distributed online and by the Rabbit Welfare Association and Fund, the survey received 1516 responses. It was found that increased owner perceptions of pain, emotions, and intelligence resulted in increased likelihood of providing a partner, increased enrichment variation, and a more appropriate diet and type of housing. Welfare scores were associated with diet, housing, variety of enrichment, and time spent with owners. These results suggest that a practical approach to improving the welfare standard provided to rabbits may be to target improving owner perceptions of the species’ intelligence, emotionality, and experience of pain. This information would be beneficial in tailoring public education programs to increase provision of welfare enhancing resources, improve the human–animal relationship, and thus improve the welfare standards for this species.

## 1. Introduction

Pet rabbits are the third most popular pet in the western world [1], however with an increasing interest in their welfare from within the veterinary profession and the welfare science community, widespread welfare deficits have become apparent [1,2,3,4,5,6]. The British Veterinary Association (BVA) noted with consternation that 78% of UK vets believe pet rabbits’ welfare needs were not being met, reporting rabbits being housed inappropriately or alone and within an inappropriate environment [1].

Several survey-based studies have been carried out within the last decade to collect data on the current state of rabbit welfare. Rooney et al. [7] ran a large resource-based survey of 1254 owners within England, finding that only 41% of owners keep their rabbit with a partnered rabbit; and Mullan and Main [4] in their survey of health and husbandry of 102 rabbits found that 44% were housed alone, only 65% of rabbits were fed greenstuffs daily (with 20% not having daily access to hay), and 20% kept in a hutch smaller than 0.54 m^2^. Both Rooney et al. [7] and Mullan and Main [4] state that this inappropriate housing, without conspecifics, and often an inappropriate diet, would limit the behavioral repertoire and welfare of pet rabbits [4,7]. This welfare deficit due to potential husbandry failings within our pet rabbit population could lead to welfare compromise, a shortened lifespan, and painful medical problems such as dental disease [3,4,5]. Welch et al. [8] carried out a large survey of 2890 rabbit owners (focused in the USA and Canada, but also covering some areas outside of North America), which focused on their knowledge of rabbit husbandry and their rabbits’ neutered status. They found that accessible education and regular contact with veterinarians had a significant influence on owner knowledge and subsequently the neutered status of their rabbits. These findings implied the potential of increasing owner knowledge for the betterment of rabbit health through preventative care and thus for the betterment of welfare.

Recently, Rioja-Lang et al. [5] gathered rabbit experts of varying demographics to produce a list of priority welfare issues requiring investigation within the pet rabbit population, which included inadequate housing, diet, handling, and socializing; a lack of preventative healthcare and a lacking of knowledge in both owners and veterinarians; all contributing to a shortened life expectancy and welfare deficit. It was suggested that working to resolve the prioritized issues should rely on an educational approach to improve owner knowledge and attitudes towards the species in order to encourage human behavioral change. It is hoped that a change in attitude of the general public towards rabbits would have a positive influence on resource provision, and thus the health and wellbeing of the species.

An appropriately enriched environment (including basic necessities for this species such as the provision of hides, items to chew on and to investigate, and open areas to run and hop) have a significant effect on welfare, as well as the behavior demonstrated between group housed individuals. For example, in a laboratory setting, provision of enrichment within a farmed rabbit setting in the form of wooden sticks to chew lead to a lower incidence of skin injuries, which could be extrapolated to pet rabbits [9]. Mullan and Main [4] suggest that the human–rabbit bond may also be strengthened with access to an enriched environment and human contact.

Recently, there has been an increasing interest in the mutual benefits of human–animal relationships, and the human attitudes towards animals that impact these relationships [10,11]. Along this line, studies have been carried out surrounding public perceptions of fish sentience [12,13] as well as sheep sentience [14] in the form of owner surveys, in order to better understand how the perceptions of these animals impact human behavior towards them. To the authors knowledge, a large-scale study exploring perception and attitudes towards pet rabbits’ intelligence, emotionality, and ability to experience pain has not yet been undertaken.

Although perceptions of rabbit pain, intelligence, and emotionality have not yet been studied, perceptions of rabbit husbandry requirements have been found to significantly affect the human–animal relationship and pet rabbits’ welfare. A small survey-based study (*n* = 52) by Edgar and Mullan [6] at the time of purchase of pet rabbits found that owner attitudes and knowledge of rabbits’ husbandry had a strong correlation to husbandry and subsequent welfare. This included deciding whether to neuter their pets, which mix to feed and whether or not to house them with a companion. Their study suggests that improving the public’s knowledge of companion rabbits may be vital in improving their welfare. This was corroborated by Tamioso et al. [14], suggesting that attributing emotionality to animals is significantly associated with more positive treatment from their owners.

There has not yet been a large-scale study regarding the public’s perception of rabbits and their mental abilities, but investigation of the subject is key to target public opinion and thus human behavioral change. By using an online survey for current rabbit owners, this study aims to provide a novel understanding of the public’s perceptions of rabbits; how owner perceptions of rabbits’ ability to experience pain, emotions, and intelligence affect the resources and husbandry that they are provided with (including the important resource of a partner); and how these various resources affect the rabbits’ welfare. Knowledge of such relationships could be beneficial in tailoring public education programs developed by charities, pet shops, and veterinary professionals to increase provision of welfare enhancing resources such as a companion, appropriate diet, and medical care, improve the human–animal relationship and thus improve the welfare standards vital for this species [6].

## 2. Materials and Methods

### 2.1. Ethical Statement

This project gained ethical approval by the University of Glasgow Medicine, Veterinary and Life Science College Ethics Committee; project code: 200190110.

### 2.2. Questionnaire Content

A link to a duplicate of the survey distributed: https://tinyurl.com/y3oabznf.

The questionnaire used was developed using a combined approach. Review and summarization of the scientific literature, alongside expert opinion elicitation, were used in the identification of suitable questions to be used in an anonymous online questionnaire.

The questionnaire was created in Microsoft Forms (@2020 Microsoft) (Microsoft, Redmond, WA, USA) in choice, open ended, and rating style formats, based on 4 sections:

Section A: Owner and rabbit demographic information—age, gender, country of residence, and rabbit community member status of owner; age, sex, breed, time owned, spay/neuter status, vaccination status, insurance status, and if the rabbit had attended the vet for a non-routine reason. Rabbit information was limited to three rabbits per respondent due to a limitation of question numbers on Microsoft forms (with a limit of 100 questions). If respondents owned more than 3 rabbits, they were instructed to input information about their three most recently acquired rabbits.

Section B: Perceptions of pet rabbits—scoring perception of rabbits’ ability to experience pain, emotions, and intelligence from 0–100 (with 0 being equivalent to an inanimate object and 100 to that of a human); open-ended questions to elaborate on perception. As a perception study based on rabbits is novel, this scale was chosen based on intelligence-based perception studies by Nakajima et al. [11] and Mercy for animals [12], Tamioso et al. [14], which used 0–100, 0–10, and 1–5 point scales, respectively). Open-ended questions allowing the participant to elaborate on their perceptions were included, as in Bertenshaw and Rowlinson [10].

This section also included five-point Likert scales on strength of agreement (from strongly disagree to strongly agree) that rabbits could form strong bonds with other rabbits, strong bonds with humans, and that rabbits were happy to live alone.

Section C: Resources provided to pet rabbits—enrichment (including toys, items to chew, hides, puzzles, and other); diet (including hay/grass, pellets, muesli, and vegetables/herbs); housing (including indoor hutch, outdoor hutch, outdoor free roam, indoor free roam, and other); and partner provision (housed alone, housed with another rabbit, or housed with another species) were presented as closed-choice questions. The number of days in the month rabbits would have access to the outdoors, access to indoors, and time spent with rabbits per day (observing them but not directly handling them) were asked as open questions (restricted to a numbered answer).

Section D: Individual behavioral frequency closed-choice questions (from never to several times daily)—‘binkies’, ‘flopping’, ‘zoomies’ (positive welfare state); bar biting, chewing furniture, over-grooming (negative welfare state); and self-grooming (neutral). These individual behaviors were described in a written format, and also shown as video examples so participants were familiar with behaviors being assessed as follows in Table 1:

Participants were required to fill in an individual behavior section for each rabbit included in Section A. Behavioral frequency questions were pre-determined to be positive or negative welfare indicators using previously established indicators [7]. These were coded into numbers according to frequency (0 = never, 1 = rarely up to 6 = daily) and added to produce positive and negative welfare scores made up of the frequencies of the three positive and three negative behaviors.

### 2.3. Distribution

The online questionnaire was distributed to current rabbit owners via a hyperlink on social media groups (Facebook, Instagram, and Twitter) as well as by email to Rabbit Welfare Association and Fund members. The link was presented with information regarding the importance of research to improve animal welfare, a note of appreciation for completing the survey and sharing it with fellow rabbit owners, and the opportunity to enter a prize draw to encourage responses. Reponses were collected between 10 March 2020 and 10 April 2020. Participants were required to certify that they currently owned a rabbit/s, were over the age of 16, and that they understood that participation was voluntary and could be terminated at any time and that informed consent was agreed.

### 2.4. Data Analysis

Following data collection, the raw data were exported from Microsoft forms into Excel for Mac Version 16.16.20; where all choice and qualitative responses were coded. Positive and negative welfare scores were created by adding the frequencies of behaviors (previously deemed positive or negative indicators of welfare) to provide a score/18. For example, if a participant reported their rabbit to ‘binky’ daily, to ‘flop’ weekly, and to ‘zoomie’ never, their positive welfare score would be calculated as 6 (daily) + 5 (weekly) + 0 (never) to give a positive welfare score of 11/18.

All variations of entry for each country were transformed. Cleaned sheets from Excel were then exported to IBM SPSS Statistics Version 26 (IBM, Armonk, NY, USA) and descriptive statistics were carried out.

Data were not normally distributed; therefore, non-parametric statistics including independent-sample Kruskal Wallis Tests, Mann–Whitney U Tests were performed as an overview of potentially significant variables to enter into multivariate statistical models. The independent variables were the perception scores for rabbits’ intelligence, emotionality, and pain level, and the dependent variables were housing, diet, partner provision, enrichment score, time spent, and welfare scores. Spearman’s Rho correlations were used to test if perception scores correlated with welfare scores, time spent with rabbits, and enrichment score. The independent variables were the perception scores for rabbits’ intelligence, emotionality, and pain level, and the dependent variables were time spent and welfare scores.

Box-Tidwell [15] procedures were used to test the assumption of a linear relationship between the continuous independent variables and the logit of the dependent variable and Bonferroni correction was applied using all terms in the model resulting in statistical significance being accepted when *p* < 0.00625 [16]. Based on this assessment, all continuous independent variables were found to be linearly related to the logit of the dependent variable. Binomial logic regressions were then run for perception and partner provision.

Multinomial logistic regressions were run for perceptions and the remaining resources: Housing, diet, and enrichment score. To test the assumption of a linear relationship between the continuous independent variables and the logit of the dependent variable, the independent variables were graphed against the logit of the dependent variables, and visually inspected for linearity. All answers were provided by closed choice answers, so no significant outliers were found.

The resources against positive and negative welfare scores were found to have a linear fit using Curve Fit in SPSS (*p* < 0.0001, R^2^ = 0.015). Backwards selection regression was used to accept or reject variables as significant, and significant resources were entered into a generalized linear model with positive and negative welfare scores. To verify the model’s assumptions, a residual analysis was performed based on deviance residuals, fitted values, q-q plots, scale-location plots, and Cook’s distance. Results were deemed significant at *p* < 0.05. Graphs were plotted using Microsoft Excel.

## 3. Results

### 3.1. Respondent Information

A total of 1516 respondents completed the survey, providing complete information for 2126 rabbits. Of owner respondents: 93.9% (1423), 5.4% (82), and 0.7% (11) were female, male, and non-binary, respectively (five responded that they would prefer not to say). The majority of respondents said that they kept their rabbit with another rabbit (59.3%, 899), whilst 5.1% (78) kept their rabbit with a different animal, and 35.6% (539) kept their rabbit alone. The majority of respondents considered themselves part of a rabbit community (89.3%, 1354), and 10.7% (162) did not. Responses were received from 42 countries with the greatest number (70.5%, 1068) being from the UK, followed by the USA (11.9%, 181) and New Zealand (6.1%, 93).

#### 3.1.1. Rabbit Information

Of 2126 rabbits, 79.8% (1697) were vaccinated, 20.2% (429) were not, 24.4% (519) were insured, and 75.6% (1607) were not. The largest groups of breeds entered were: 27.0% (574) of rabbits crossbred, 19.4% (412) mini lop, 11.0% (234) lionhead, 8.8% (187) standard lop, and 23.2% (493) unknown breeds.

#### 3.1.2. Resources Information

**Figure 1:**
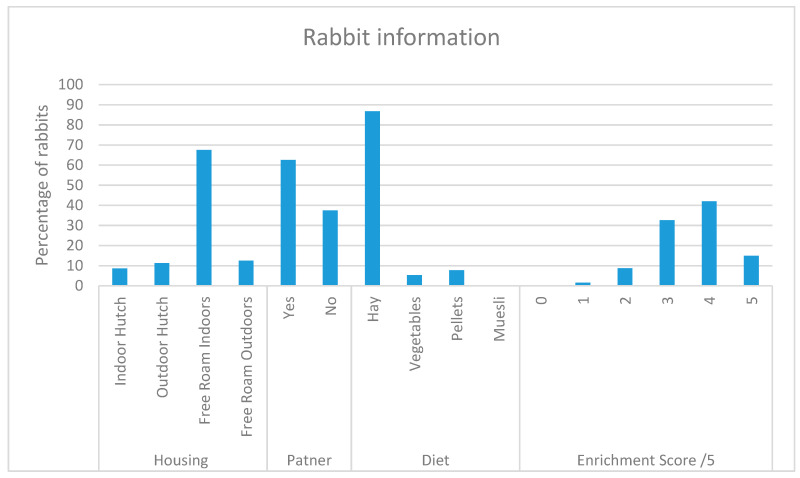
Answers provided to “where does your rabbit spend most of its time”; “Is your rabbit kept with a partner/partners?”; “what makes up the majority of your rabbit’s diet (over 50%)?”; “Which of the following is your rabbit provided with?” *n* = 1516.

The majority of participants in this study kept their rabbit in free roam indoor housing, provided a partner to their rabbit, fed a diet of a majority of hay, and provided four different items of enrichment (Figure 1).

#### 3.1.3. Perception Information

The median intelligence, emotions, and pain that participants perceived rabbits to experience were 70, 90, and 100, respectively, out of a total of 100 (Table 2). The majority strongly agreed that rabbits could form strong bond with other rabbits; and could form string bonds with humans. The majority of participants neither agreed nor disagreed that rabbits were happy to live alone (Table 3).

### 3.2. Do Perception Scores Correlate with Welfare Scores?

A Spearman’s Rho correlation found a positive correlation of the ranks of perception scores for intelligence, emotions, and pain with positive welfare score, time spent per day, and enrichment score, and a negative correlation of the ranks of perception scores for intelligence, emotions, and pain with negative welfare score (Table 4).

### 3.3. Does Perception Score Have a Relationship with Resources Provided?

Logistic Regressions.

#### 3.3.1. Partner

A binomial logistic regression was performed to ascertain the effects of perception of rabbit’s ability to experience intelligence, emotions, and pain on the likelihood that participants house their rabbit with a partner. The logistic regression model was statistically significant, *χ*^2^(4) = 27.402, *p* < 0.0001. The model explained 0.8% (Nagelkerke R^2^) of the variance in partner provision and correctly classified 62.5% of cases. Of the three predictor variables, only two were statistically significant: Increasing perception of emotions (Exp(*B*) = 1.005, *p* = 0.016) and pain (Exp(*B*) = 1.004, *p* = 0.028) were associated with an increased likelihood of providing a partner.

#### 3.3.2. Housing

A multinomial logistic regression was run to predict housing provided to pet rabbits according to perception scores for intelligence, emotions, and pain. The model fit against the intercept significantly (*p* < 0.0001). The model predicted that participants were significantly more likely to provide free-roam indoor housing and free roam outdoor housing compared to an outdoor hutch with a higher pain perception score (Exp(*B*) = 1.015, *p* < 0.0001 and Exp(*B*) = 1.017, *p* < 0.0001, respectively), but not with intelligence nor emotion score.

#### 3.3.3. Diet

A multinomial logistic regression was run to predict type of diet provided to pet rabbits according to perception scores for intelligence, emotions, and pain. The model fit against the intercept significantly (*p* < 0.0001). The model predicted that participants were significantly more likely to provide a diet of majority vegetables compared to muesli with a higher intelligence perception score (Exp(*B*) = 1.048, *p* = 0.019).

#### 3.3.4. Enrichment

A multinomial logistic regression was run to predict the amount of enrichment provided to pet rabbits according to perception scores for intelligence, emotions, and pain. The model fit against the intercept significantly (*p* < 0.0001). The model predicted that participants were significantly more likely to provide a greater variety of enrichment with a greater pain perception score. Compared to providing no enrichment the likelihood of providing two different forms of enrichment (Exp(*B*) = 1.049, *p* = 0.011), three different forms of enrichment (Exp(*B*) = 1.058, *p* = 0.003), four different forms of enrichment (Exp(*B*) = 1.054, *p* = 0.005), and five items of enrichment (Exp(*B*) = 1.062, *p* = 0.002) all increased with increasing pain perception score.

### 3.4. Which Resources Impact Welfare Score?

Generalized Linear Models.

Backwards selection was used to remove non-significant (*p* > 0.05) variables from the model. With regards to positive welfare score, housing, neutered status, and partner were removed as they were not found to have a significant relationship, whilst partner and diet were removed with regards to negative welfare score.

#### 3.4.1. Positive Welfare Score

The GLM had a good fit (*χ*^2^ = partner 248.37, *p* < 0.0001 suggesting that the observed data corresponded to the fitted model), and found a positive welfare score had a very strong positive association with a majority grass/hay diet (*B* = 6.588, *p* = 0.003), majority vegetables diet (*B* = 6.240, *p* = 0.005), and a majority homogenized pellet diet (*B* = 5.290, *p* = 0.018) compared to a majority muesli diet. A positive association was found with positive welfare score and enrichment score (*B* = 0.735, *p* < 0.0001) as well as with time spent with pet rabbits (*B* = 0.005, *p* < 0.0001) (Table 5).

#### 3.4.2. Negative Welfare Score

The GLM had a good fit (*χ*^2^ = partner 85.37, *p* < 0.0001), and found negative welfare scores were strongly positively associated with outdoor hutch housing when compared with outdoor free roam housing (*B* = 1.857, *p* < 0.0001). Negative welfare score was also positively related to housing in an indoor hutch, compared with free outdoor roam (*B* = 0.689, *p* = 0.01); and a weak positive association was found between negative welfare score and time spent (*p* < 0.0001, *B* = 0.002). Negative welfare score was significantly negatively associated with a higher enrichment score (*B* = −0.193, *p* = 0.011) Table 5.

### 3.5. Open Ended Responses

Elaborated responses to “If you would like to comment on rabbits’ intelligence, emotions and pain, please do so briefly here” are provided in Table 6, Table 7 and Table 8. Responses were coded if the same words or their synonyms were stated. Direct quotes that the authors felt most represented the statements being made by multiple participants were chosen.

When elaborating on rabbit intelligence, the most frequent mentioned terms included problem-solving abilities, recognizing owners, communication through behavior, choosing whether to follow commands or not, the variation in individuals, awareness of time and routine, knowing their name, and the ability to be trained. It was also frequently mentioned that rabbit intelligence is underestimated/misunderstood (Table 6).

When elaborating on rabbit emotion, the key comments included that rabbit emotion was obvious, that rabbits express emotion through body language and behavior, that they show fear, they grieve, they show sadness, experience happiness, visibly express joy, and ‘binky’ (see Table 1 for behavior description) when happy (Table 7).

When referring to rabbit pain, the main elaborations included: ‘rabbits have different anatomy so feel pain differently’; ‘all animals feel pain as humans do’; ‘you can tell behaviorally’; ‘it’s obvious, emotional and physical pain’; ‘as fully as humans’; ‘physical pain’; and ‘doesn’t express pain the same’. Many responses mentioned that rabbits hide their pain as a prey species (Table 8).

## 4. Discussion

This novel study, a large survey of 1516 owners, obtained valuable information on the current state of perceptions of rabbits’ mental abilities, how such perceptions influence resource provision, and which of these resources most impact the welfare of pet rabbits. Choice questions regarding resource provision excluded explicit details such as measurements of enclosures (as included in previous rabbit surveys [4,7] in order to understand the current state of resource provision of owners to their pet rabbits). Instead, the purpose of this section was to analyze the indicators that lead to owners providing them with various general resources (such as diet, housing, enrichment, etc.). The knowledge gained from this work could be valuable in tailoring public education via pet care professionals, charities, and veterinarians to target improving perceptions, and subsequently the welfare of the species; as well as in informing future studies on the objective mental abilities of rabbits.

### 4.1. Summary of Results

A positive correlation of ranks was found between perception scores with positive welfare score, time spent per day, and enrichment score; and a negative correlation was found between perception scores and negative welfare score. Participants with higher emotion perception scores were more likely to provide a partner; participants with higher pain perception scores provided a higher enrichment score and were more likely to provide free roam housing compared to hutched housing; participants with higher intelligence perception scores provided a more appropriate diet. Positive welfare score had a very strong positive association with a majority grass/hay diet compared to a majority muesli diet and had strong positive association with a higher enrichment score. Negative welfare score was positively related to housing in a hutch compared with free-roam housing and was negatively related with an increased enrichment score.

### 4.2. Perceptions of Rabbits

#### 4.2.1. Intelligence

Intelligence is a subjective notion, ill-defined within our own species, and often applied anthropocentrically to other species. Bekoff [17] highlights a link between an animal’s perceived ability to think, and the subsequent treatment of that animal species by humans. Indeed, Smuts [18] states that the stage before establishing a mutually beneficial interspecies relationship is the acknowledgment of a fellow social being with which communication is possible. Kirkwood [19] highlights the moral responsibility to consider the feelings of species that have cognitive processes such as our own, and the subsequent need to re-evaluate our treatment of animal species. This sense of ‘mind’ is commonly seen in reference to the human ability to think, and not in a species-specific format. As information on owner perception of pet rabbits had not previously been gathered, this study used peer reviewed past literature of the perceptions of various domesticated animals to form the design of the perception section of the survey. Intelligence, emotions, and pain felt by rabbits was compared to that of humans on a 0–100 scale, with 0 being equivalent to that of an inanimate object, and 100 to that of a human. It is acknowledged that this anthropocentric format of assessing intelligence may require species-specific readjustment to assess the true objective intelligence of a species. However, as the objective of this work surrounds the hypothesis that attribution of mentality to rabbits would shape owner behavior towards improved resource provision (through a hypothesized improved level empathy towards the animal), a human-centric approach is felt to fit this line of study. This scoring indeed would not tell us how intelligent rabbits are, but how intelligent owners perceive them to be.

Participants of this study scored rabbit intelligence with a mode of 80/100, and a mean 69.97/100; making it the lowest ranked perception of rabbits when compared to emotions or pain.

Literature investigating the true intelligence of rabbits is limited. A comparative intelligence test study by Livesey [20] involving rabbits, cats, and rats found rabbits to be ‘slower’ than cats and rats, and “froze” frequently. However, the author concluded that the Hebb and Williams test performed should be used and interpreted with considerable caution, especially if used to compared different species. Six percent of participants in this study emphasized the individual variation even within a species, which indeed should be an important factor in considering intelligence [17]. Further species-specific testing of intelligence would need to be carried out to measure the intellectual capacity of rabbits in order to relate true intelligence to perceived intelligence.

The behaviors and attributes included in elaborated answers by the participants of this study in relation to rabbit intelligence could serve to inform those intent on testing rabbit intelligence. For example, 10% of owners described their rabbits to be very accurate with time and routine, 21% said they could be trained easily, and 6.5% claimed their rabbits could solve puzzles. Perhaps most interestingly, 5% experienced their rabbit choosing or deciding whether to follow training or not; and 4% claimed their rabbits actively deceive their owners in order to reach areas deemed specifically as ‘no entry’ through distracting behaviors and displacement, which, if proven, would indeed suggest high levels of intelligence within this species [10]. Many participants mentioned the misunderstood nature of rabbit intelligence, requiring an adequate environment and degree of experience with rabbits to ‘unlock’.

#### 4.2.2. Emotions

The emotion scores of rabbits had a mean of 83.78/100, and a mode of 100/100, indicating that owners believe their rabbits are capable of emotional affective states. It has been demonstrated that gender affects attitudes towards animals with females showing more positive behaviors and attitudes than males [21,22]. It should therefore be considered that as the participants of this study were a majority female, the mean and mode scores for emotion may have been different in a study with more evenly spread participation across genders, reducing these results’ generalizability.

Bertenshaw and Rowlinson [10] and Martens et al. [23] argue that owners’ and farmers’ have a more accurate appraisal of their animals’ emotions compared to non-owners, and that positive emotions were more readily recognized. For example, Martens et al. found an increase of perceived attunement/mirroring of emotions with pet dogs with increasing attachment, similar to the relationships experienced within human-human relationships [23].

Open-ended questions allowing the participant to elaborate on their perceptions were included, as in Bertenshaw and Rowlinson [10], to provide further evidence for discussion and to provide the opportunity for new hypotheses to be generated. It was often mentioned by participants that they could tell how their animal was feeling by looking at their face or their eyes, or by their behavior. However, as animal welfare studies move towards trying to understand welfare from the animal’s perspective, a key concern is anthropomorphism [24]. Due to the purely subjective nature of emotion, the presence and complexity of emotions felt by non-human animals is still fiercely debated within the scientific community [25]. The difficult topic of anthropomorphism versus increased perception of emotionality should be further investigated to come towards a vital level of agreement and perhaps guidelines for interpretation.

The participants of this study suggest that their rabbits experience both primary (e.g., fear, joy) and secondary emotions (e.g., anger, jealousy), similar to those attributed by owners to cats and dogs in a study by Martens et al. [23]. However, participants in this study frequently noted that interpretation of emotion requires experience with their animal to interpret the often-subtle behaviors that indicate said emotions. It should be noted that elaborations of emotions contained the largest range of responses when compared to intelligence and pain, indicating the complexity of potential emotional experience and the interpretation of it.

It is widely accepted that mammals, as sentient beings, experience basic evolutionarily beneficial emotions such as fear and pleasure [25]; but further work would need carried out to confirm the presence of more complex secondary emotions in rabbits. Panksepp and Watt [26] define basic emotions as “seeking, fear, rage, lust, care, panic, and play”, which serve to stimulate punishment or reward in the limbic system for learning in response to behaviors. They state that “more subtle emotional feelings such as jealousy, shame, guilt or a sense of humor—feelings that are created by the interrelations of basic emotions with higher cognitive processes—remains an open issue”, all of which were mentioned in the elaboration of emotions by the participants in this study [27]. Whilst the subjective affect felt by rabbits is not known, it is clear that within the literature the attribution of emotionality to animals leads to welfare improvement.

Work by Tamioso et al. [14] concluded that attributing emotionality to animals is significantly associated with more positive treatment from their owners; Bertenshaw and Rowlinson [10] found dairy farmers that demonstrated positive attitudes towards animals had a significant relationship with yield and human–animal relationship; and Martens et al. [23] found that an attribution of higher emotionality led to an improved human-animal bond score in dogs and cats.

Bekoff [17] suggests subjective assessments of animals should be considered objectively just as “supposedly objective scientific facts”; and due to the great uncertainty surrounding the inner lives of animals, they should be given the benefit of the doubt. The impartment of emotions to pet rabbits by the participants in this study, whether anthropomorphic or an accurate interpretation, correlated with a higher positive welfare and lower negative welfare score. This result is in keeping with the aforementioned conclusions of previous literature that a higher perception of animal emotion leads to better treatment, and better subsequent welfare. The true emotions felt by all non-human animals remains up for debate to shape animal ethics, but perhaps a confirmed answer is not a necessity practically if it is repeatedly shown that perceiving emotionality improves animal welfare. Thus, education of new or existing owners about the emotional capabilities of rabbits is likely to be of benefit for domestic rabbit welfare.

#### 4.2.3. Pain

The participants in this study rated rabbits’ ability to experience pain in comparison with humans very highly, with a mode of 100/100. However, answers did range from 0 (the equivalent of that of an inanimate object) to 100 (equivalent of a human), with a mean of 89/100. The key notion mentioned by 26.56% of 800 participants that elaborated on rabbits’ ability to experience pain was that as prey animals, they hide their pain. This indeed makes pain assessment challenging in its subtly and could lead to a misunderstanding that rabbits do not or are not experiencing pain.

There is strong evidence to suggest vertebrates experience pain as of their anatomy and physiology, as well as their behavioral reactions to noxious stimuli [28,29]. Foley et al. [29] state that small research animals (such as rabbits) undoubtedly centrally process pain equally to other large animals such as cats, dogs, and primates.

However, the gold standard for reporting pain in humans remains as self-report. This leaves non-verbal animals, and human infants’ experience of pain, to debate within the scientific community.

It has been shown across many laboratory animal pain studies that small mammal pain is often underestimated and under-dosed, leading to a significant welfare impact [29,30,31]. If trained veterinarians and experts in small laboratory mammal physiology are underestimating pain in their subjects, one can only imagine the deficit in knowledge and assessment in pain of the pet rabbits owned by the general public. This suggests that the public, and indeed vets and licensed animal care providers, require better education and knowledge of the subtle pain signs shown by rabbits in order to improve welfare of the domesticated species.

### 4.3. Perception Affects Welfare (Intelligence, Emotions)

Bekoff [17] discusses how perceptions of consciousness and cognition of non-human animals informs treatment and moral stance, and thus welfare. Kılıç, İ. and Bozkurt [22] found that in sheep, not only did a higher perception level of animal welfare lead to higher welfare standards on farm, but that the attitude of emotions and cognition in sheep helped to shape these welfare perceptions.

Bertenshaw and Rowlinson [10] found dairy farmers that demonstrated positive attitudes towards animals had a significant relationship with yield and human–animal relationship. Similarly, Hemsworth et al. [32] found farmers with more positive perceptions of cows had animals that expressed less fear-associated behaviors and had higher production yields. A small survey-based study by Edgar and Mullan [6] of 52 owners at the point of purchase of pet rabbits found that knowledge and attitudes surrounding the species were indeed significant factors in husbandry and resource provision such as providing a companion, an appropriate diet, and an intention to neuter/spay their new pet.

Changing perceptions of the emotional and intellectual capacity of rabbits could therefore be key in changing human behavior towards improving the standard of welfare provided to this species. This is corroborated by Tamioso et al. [14], suggesting that attributing emotionality to animals is significantly associated with more positive treatment from their owners.

In this study, there was a significant positive relationship between perception scores for the intelligence, emotions, and pain experienced by rabbits and time spent with rabbits (without directly handling them), and also with levels of enrichment.

These results suggest that perceptions of rabbits may influence their welfare in both a negative and positive sense, so warrant further work within public engagement to further educate the public about rabbits’ ability to experience these elements of sentience. The amount of time a person spent directly observing their rabbits, and the enrichment they provide their rabbits with, seemingly directly influenced their perceptions: Perhaps through the witnessing of more behaviors that imply intelligence and emotionality.

### 4.4. Perception and Improved Welfare Practical Changes

The results of this study highlight the great importance of owner perception of rabbit mentality to improve welfare. It is therefore suggested that public education be tailored to improve the perception of rabbits’ ability to experience, and to promote empathy towards the species. Notable behaviors were mentioned by participants in this study that greatly impacted them and were spoken of very emotively. Perhaps these behaviors could be used to cause an emotional impact on new owners and alter their perceptions of rabbits. For example:

- “Binkies” and “zoomies” were seen to be an unequivocal sign of joy in pet rabbits by owners, and it was repeatedly reported to also be a great joy for owners themselves to witness.
- Vocalizing in pain in rabbits was spoken of extremely emotively by those that described it, making pain an unquestionable experience of rabbits to those owners. They spoke of it being ‘unforgettable’ and ‘heart breaking’.
- The bond between rabbits that had been successfully group housed was spoken of as indescribably strong, and frequently described as ‘love’.
- The perceived grief experienced by rabbits was frequently mentioned when attributing emotional pain, emotionality, and emotional intelligence, and had a great impact on owners that witnessed it, highlighting the strength of the intraspecies bond.
- The ability of rabbits to learn tricks, their perceived ability to understand timing and routine, and their spatial memory had clear impact on owner perception of rabbit intelligence.

As discussed, the roll of pet care professionals and veterinarians in face-to-face education at the point of purchase and onward throughout the life of rabbits has an impact on health care level and resources provided to these animals. Perhaps this public education should not only be provided through rabbit associated campaigns, but also shown to vets and pet care professionals, altering their own perception of rabbits, and thus perhaps influencing them to push for better standards from owners.

### 4.5. How Welfare Relates to Resource Provision

It was found that housing, neutered status, and partner did not have a significant impact on positive welfare scores, whilst partner and diet had no impact on negative welfare scores.

It is widely accepted by key rabbit organizations (such as the Rabbit Welfare Association and Fund, as well as veterinary organizations such as the BVA), that for adequate social interaction and performance of natural behavior, rabbits should be kept in groups of at least two individuals. However, there is very limited published literature on the single or group housing of rabbits within a pet rabbit setting from which to extrapolate an expected positive welfare impact of group housing.

Some studies regarding group housing of rabbits in a commercial setting include Szendro et al. [33] found that group housing increases aggression, stress, injuries, and mortality whilst reducing production and shortening lifespans, resulting in the advice that commercially housed rabbits should be housed singly. Perez-Fuentes et al. [2] also reported group housed commercial does have increased cull percentages, increased cortisol levels (indicating an increased stressed state), and increased kit mortalities. These studies show a pronounced negative effect on both the welfare of does and of productivity when rabbits are housed in groups, presenting an argument to the otherwise accepted notion that rabbits should be kept in conspecific groups. It is however worth arguing that placing six entire rabbits aged 19 weeks during pregnancy [2] and entire 17-week-old female rabbits that had been previously housed individually into group housing [33] within a commercial setting’s caged environment is not directly comparable to the majority of pet rabbit housing in many ways. For example, commercial rabbit housing generally consists of entire does, often involves gestation and production of kits, and the caged housing systems that the does are kept in are very different to the majority of companion animal rabbits.

Animal bonding is defined by Abrantes [34] as a biological connection formed between animals to form a connection that is thought to promote cooperation. Bonding rabbits is anecdotally known be a slow and intensive progress when bonding two young and neutered individuals, so it can almost be seen as unsurprising that injuries and mortalities would increase when placing these individuals together under the circumstances outlined by these group versus single housing studies by Perez-Fuentes et al. [2] and Szendro et al. [33].

Other negative associations were found in rabbit studies; reporting antagonistic behaviors displayed in 78% of rabbits housed with companions [4]; 25% of group housed rabbits reported to fight at least occasionally [7]; and group housed rabbits displaying more fear when placed in a new environment than singly cage housed groups [35].

Contrary to the studies outlining the welfare concerns of group housing rabbits, Bozicovich et al. [9] found that group housed does within a commercial setting (that were provided with enrichment and grouped at 35 days old) demonstrated the lowest incidence of aggressive behavior. Mixed gender groups were shown to have an increased incidence of social interactions and a lower incidence of stereotypies [9]. This study advised, therefore, that mixed gender groups should be housed collectively from weaning at four weeks of age.

Trocino et al. [35] reinforces the findings towards the benefits of group housing, reporting a more complete behavioral pattern and a heightened boldness towards humans when commercial rabbits were housed in groups. It is suggested that not all pairs of rabbits are compatible, presenting a possible significant welfare issue with keeping some rabbits in groups.

Within a pet rabbit setting, surveys first by Mullan and Main [4], found that all owners reported their group housed rabbits to display agonistic behavior, 80% playing together, and are often observed by owners to have a strong bond. A larger study by Rooney et al. [7] reported rabbits living with conspecifics were reported to rest, groom, and play together ‘very often’. A recent study of UK shelter-housed pet rabbits found pairs to have reduced stress, improved thermoregulation, and no witnessed bar biting when compared to singly housed rabbits [36].

A highlight of the literature surrounding the group housing of rabbits is that evidence is scant for pet rabbit owners, and those that inform them (such as veterinarians and pet care advisors). Future work regarding the group housing of rabbits in a pet setting, and perhaps the characteristics of pet rabbits that form the most reliably strong bonds could be a step forward in advising owners: In order to reduce rehoming and injury, and to improve the longevity and success of rabbit bonding.

### 4.6. Methodological Consideration

In this study, data were collected from 1516 participants and fully completed information was provided for 2126 individual rabbits. Survey links were posted on rabbit social media forums and emailed out to members of the Rabbit Welfare Association and fund. This would have likely skewed the data collected towards both particularly enthusiastic rabbit owners, and rabbit owners with time and access to the internet [7]. Indeed, 89.3% of participants considered themselves part of a rabbit community, 93.9% of participants were female, and 70.5% from the UK.

Whilst the skewed population of participants carried a degree of variation in perception scores and in resources provided to analyze their relationship, the responses cannot be generalized to that of the entire rabbit owning population. It must be acknowledged that rabbit enthusiasts may indeed think more highly of their pet’s mental ability, impacting the response figures received. It should also be noted that the authors are also females from the UK within the veterinary profession, which will have had an impact due to bias on the study design.

It has been posed in previous survey-based studies that online surveys would skew data by age group, with only younger groups computer literate enough to access the survey [7]. However, our online survey showed a fairly even proportion of participation between age groups over 20 years old (5.2% under 20, 35.1% 20–30, 23.3% 30–40, 21.1% 40–50, and 15.3% over 50 years). As time moves on and more age groups become daily users of computers and social media, perhaps reconsidering online surveys as a possibility to receive data from a range of age groups should be highlighted.

As participants were self-reporting information about their rabbits, there may be a discrepancy between stated resources and actual resource provision. Due to logistical constraints, this study did not perform home visits to assess resources or rabbit behaviors. Rooney et al. [7] specified a need for further research into the accuracy of reporting in surveys. An example of this in the Mullan and Main [4] rabbit resource study was that 91 participants stated their rabbits received hay daily, but hay was only available to 83 at the time of assessment. As our survey was purely anonymous (and participants were aware that their email addresses were not associated with their responses), it may be considered that inaccurate reporting would be minimized due to the lack of pressure to provide socially preferred answers [23].

Dewitt et al. [37] states internet-based research as “advantageous to essential” in recruiting niche communities; but that it also brings forth a new validation issue that the researcher will never meet their participants. This can lead to the same participant completing the survey multiple times and ineligible participants completing the survey. Santesso et al. [38] suggest that strong conclusions should be avoided in studies with a low participation rate due to the potential inaccuracies and biases of participation within surveys. This study gained a large response rate of 1516 participants, but the discussion and conclusions drawn from the results of this study should take respondent bias into consideration.

In future studies, home visits should be considered in order to validate the data provided in responses on resources provided and frequency of rabbit behavior.

### 4.7. Further Work

- Notable anecdotal behaviors that imply intelligence and emotionality in this species were shared in open-ended questions that require further investigation to objectively assess rabbit intelligence and emotionality in an objective and species-specific format.
- Future studies focused on the public’s perceptions of rabbits should aim to receive responses from a more even distribution of owners in terms of gender, country of origin and of rabbit community status to allow for generalization of results.
- A study showing the impact of perception-targeted public education on resource provision and welfare of rabbits would be useful to confirm the advice arising from the results of this paper that improving perception scores may improve welfare.
- Work should be carried out regarding the characteristics of rabbit groups that produce the most successful bonds, with the highest incidence of agonistic and lowest incidence of antagonistic behaviors.

## 5. Conclusions

Higher perception scores of intelligence, emotions, and pain provided by owners were consistent with higher positive welfare scores. This suggests that a practical approach to improving the welfare standard provided to rabbits by owners may be to target improving these perceptions of the species’ features of sentience. These may be directly targeted through public engagement: Demonstrating behaviors of rabbits that infer such qualities or improved through encouraging owners to spend more time with their rabbits, and to provide them a greater range of enrichment, shown in this study to relate to perception scores.

It is thought this perception-centric approach could be essential to hone in on improving empathy and compassion towards this species, deemed widely as an inexpensive and replaceable ‘child’s pet’; to move towards rabbits being seen as a sentient, sociable mammal with a wide range of complex and dynamic behaviors that deserve respect and a high welfare standard.

## Figures and Tables

**Table 1:** Descriptions provided to participants describing behaviors.

Behavior	Description	Behavior	Description
“Binkies”	“Spontaneous leaps into the air, sometimes with body twists” https://youtu.be/h42tonjL-SE	“Bar Biting”	Scratching or biting at bars of hutch (if applicable)
“Zoomies”	“Fast, excited running that doesn’t involve chasing to mount/bite”	“Chewing Furniture”	Chewing the furniture or hutch
“Flopping”	“Flopping onto their side” https://youtu.be/uIZJgC42Cfs	“Over-Grooming”	The rabbit has noticeable bald patches on its body, usually around the belly

**Table 2:** Answers provided to “The ability of rabbits to experience emotions (e.g., sadness, joy, fear), intelligence, and pain the same way humans do”, between 0 and 100.

Perception	Median/100
Intelligence	70
Emotions	90
Pain	100

**Table 3:** Answers provided to “Please choose how much you agree or disagree with the following statements: Rabbits can form strong bonds with other rabbits; Rabbits can form strong bonds with humans; Rabbits are happy to live alone” *n* = 1516.

	Strongly Disagree	Disagree	Neither Agree nor Disagree	Agree	Strongly Agree
“Rabbits can form strong bonds with other rabbits”	3.4% (52)	0.5% (7)	2.4% (36)	9.4% (142)	84.4 (1279)
“Rabbits can form strong bonds with humans”	2.6% (40)	0.3% (4)	2.4% (37)	27.0% (409)	67.7% (1026)
“Rabbits are happy to live alone”	26.6% (404)	28.6% (435)	29.1% (441)	12.8% (194)	2.8% (42)

**Table 4:** Spearman’s Rho Correlation matrix (*n* = 2126), Ns = *p* > 0.05.

	Intelligence	Emotion	Pain	Enrichment	Time Spent	Positive Welfare	Negative Welfare
Intelligence	-	0.477 *	0.200 *	0.106 *	0.135 *	0.185 *	−0.047 *
Emotion	0.477 *	-	0.442 *	0.116 *	0.129 *	0.123 *	−0.064 *
Pain	0.200 *	0.442 *	-	0.101 *	0.108 *	0.064 *	−0.147 *
Enrichment	0.106 *	0.116 *	0.101 *	-	0.210 *	0.249 *	Ns
Time Spent	0.135 *	0.129 *	0.108 *	0.210 *	-	0.269 *	Ns
Positive Welfare	0.185 *	0.123 *	0.064 *	0.249 *	0.269 *	-	0.115 *
Negative Welfare	−0.047 *	−0.064 *	−0.147 *	Ns	Ns	0.115 *	-

* indicates *p* < 0.05.

**Table 5:** Results from generalized linear model for housing, diet, enrichment score, neutering, partner, and time spent with positive and negative welfare score.

Variables	Mean Response	*B*	Error	*p* Value
Positive Welfare Score (R^2^) = 0.086				
Diet				
Hay/Grass	14.41	6.588	2.216	0.003
Vegetables	13.74	6.24	2.2388	0.005
Pellets (homogenous)	13.84	5.29	2.2316	0.018
Muesli	7.00	Ref		
Enrichment Score		0.735	0.08	<0.0001
Time Spent		0.005	0.0005	<0.0001
Negative Welfare Score (R^2^) = 0.065				
Housing				
Outdoor Hutch	7.38	1.857	0.2927	<0.0001
Indoor Hutch	6.11	0.689	0.291	0.01
Indoor Free Roam	5.70	0.171	0.2083	NS
Outdoor Free Roam	5.37	Ref		
Neutered Status				
Neutered	5.74	0.558	0.1901	0.003
Not Neutered	6.34	Ref		
Enrichment Score		−0.193	0.0764	0.011
Time Spent		0.002	0.0005	<0.0001

**Table 6:** Open-ended responses referring to rabbit intelligence. Statements included in the table if they were mentioned in over 3% of responses. *n* = 670.

Example Quote	Frequency	Percentage of Responses
“Rabbits are very smart, can learn tricks, can be litter trained”	217	20.79
“My rabbit seems to be aware of the time - knows exactly when she’s meant to be fed and she stamps her foot and grunts at the bowl when it’s late.”	108	10.34
“knows her name”	66	6.32
“They communicate through actions and behavior”	64	6.13
“Our Dutch rabbit is a lot brighter than the giant. Have always found intelligence varies”	63	6.03
“much more intelligent than most people realize”	61	5.84
“They know commands, but only if they choose to. Kind of like cats do.”	51	4.89
“Recognizes owners”	41	3.93
“The two are not comparable, different species and probably a lot we don’t yet know”	40	3.83
“I stop her doing something she eventually figures out a way around it”	38	3.64
“ruled by instinct”	32	3.07

**Table 7:** Open-ended responses referring to rabbit emotion. Statements included in the table if they were mentioned in over 3% of responses. *n* = 578.

Example Quote	Frequency	Percentage of Responses
“Very good at expressing happiness!”	147	9.75
“I know when my rabbit is stressed, happy, scared by her behavior & body language”	115	7.63
“When my rabbit lost his first bonded partner he grieved- very withdrawn, depressed, poor appetite”	108	7.17
“She shows fear”	105	6.97
“binkying when they’re happy”	101	6.70
“It’s obvious when they are happy, sad, bored, content”	89	5.91
“I have seen them display sadness”	75	4.98
“Rabbits clearly experience joy”	71	4.71
“My two rabbits show each other love”	50	3.32
“I can tell when my rabbit is excited”	46	3.05

**Table 8:** Open-ended responses referring to rabbit pain. Statements included in the table if they were mentioned in over 3% of responses. *n* = 505.

Example Quote	Frequency	Percentage of Responses
“as they are prey animals, they are very good at hiding pain”	213	26.56
“They definitely feel pain - you just have to know how to spot it in their behavior”	110	13.72
“Obviously!”	77	9.60
“I do believe they experience it as fully as we do”	70	8.73
“I believe all animals feel pain in the same way that humans do”	58	7.23
“Definitely able to experience and display physical pain”	42	5.24
“I believe rabbits experience both emotional and physical pain”	37	4.61
“she doesn’t express it in the same way as a human would”	37	4.61
“With very different anatomies it makes sense they’d feel pain differently.”	35	4.36
“the horrifying screaming is something impossible to forget”	27	3.37
“Highly sensitive animals”	26	3.24
